# Cases of Dysentery, Treated with Calomel in Scruple Doses

**Published:** 1817-03

**Authors:** Mauritius Power

**Affiliations:** Surgeon, R. N.


### *Cases of Dysentery, treated with Calomel in Scruple Doses.*

By Mauritius Power, Surgeon, R. N.

The first case was one of singular severity and aggravation, the cause of which will probably appear in the narrative.

Gulph of Porea, West Indies.

Case 1. Dec. 17, 1815. Samuel Collins, ætat. 19; tall, robust, and of a sanguine temperament; applied to me this morning, complaining of nausea, pain in his bowels, and frequent calls to stool. Passes blood and slimy mucus with much straining. To take a scruple of calomel and a quarter of a grain of opium twice a day.

Dec. 18. Has spent a bad day and night, with frequent purging of blood and mucus. To take the calomel and opium thrice to-day.

19th. No better. To take ℥jß ol. Ricini statim; the calomel and opium at bed time.

20th. Inspected the stools this morning. There is no blood in them, but a great quantity of greenish yellow bile and sordes with streaks of a grass green colour. To take the calomel and opium twice a day. Tea and barley water for common drink.

21st. Had frequent vomiting during the night. Blood has again appeared in the stools, with a whitish pultaceous matter. His gums are a little sore to-day, but nothing like ptyalism, though he has taken eight scruples of calomel. A scruple immediately without opium, and in half an hour a brisk dose of Castor oil. At twelve o'clock today he had an enormous stool, which filled a bed pan completely, and was composed of greenish yellow sordes, in which were five teretes, dead. One of these worms was nine inches long, and as thick as my little finger; one six inches long, and as thick as a goose quill. The others were something smaller than the latter. — Query, Have these been killed by the calomel?

22d. Spent the remainder of yesterday quiet, and took ℈j of pulv. ipecac. c. at bed time. Had some sleep and immunity from pain in the beginning of the night, but towards morning the dysenteric symptoms returned with increased violence, the stools having an exact resemblance to *lotura carnium,* intermixed with a shreddy substance. The mouth is sore, but still no ptyalism. The calomel and opium to be taken thrice to-day. To have arrow root for food, and the abdomen to be well swathed with a flannel roller.

23d. No alteration. The medicine to be continued.

24th. The mouth is now getting sorer, but the motions this morning are still like *lotura carnium* To take a scruple of calomel without opium. At four o'clock had several feculent stools. To continue the calomel and opium.

26th. Ptyalism; dysentery gone; medicines omitted. He convalesced rapidly, and soon returned to duty.

The quantity of calomel here taken might astonish those who do not know with what freedom it may be exhibited in tropical dysentery.

Case 2. Trinidad, Dec. 26, 1815. Mr. Zilkin, boatswain; rather robust, of a good constitution, and twenty-nine years of age. He was seized last night with violent pain in his bowels, followed by almost incessant purging and tenesmus; nothing but mucus and blood coming away. Such are the depression of spirits, sunk countenance, and prostration of strength, that he appears as tho'

he had been a month ill. To take ℥jß ol. Ricini immediately.

27th. Had several stools yesterday evening after the Castor oil, consisting of green sordes and bilious depraved intestinal secretions. He was relieved for a few hours; but in the night the dysenteric symptoms returned with terrible violence, and blood passes in alarming quantity. A scruple of calomel and a quarter of a grain of opium to be taken three times a day; and a flannel roller to be applied to the abdomen.

28th. Slept five hours last night, but the griping, purging, and tenesmus are most violent this morning. The depression of spirits, anxiety of countenance, and debility, are, however, mitigated. The calomel and opium to be continued as yesterday.

31st. He took three scruple doses every day since last report. His mouth is fully sore. The stools are perfectly fæculent; the griping is gone, as is also the tenesmus; in short, he is convalescent, and requires no more medicine. He returned to duty on the 9th of January, 1816.

Case 3. William Rogers, ætat. 26, of middle stature, robust, but of a melancholic temperament, was seized at the very same hour as Mr. Zilkin, with symptoms so precisely similar, that it is quite needless to repeat them. He was treated exactly in the same manner as the former patient; and as soon as ptyalism appeared, there was a total revolution in the train of symptoms, and convalescence rapidly ensued. This man passed three lumbrici, dead.— He returned to duty on the 13th of January, 1816.

Six other cases, of equal violence as the above, occurred at this time, and no other variety of treatment was pursued, except that in one case venæsection was had recourse to. In every instance of dysentery here, the calomel, in scruple doses, brought on ptyalism in a short time, except in the first case related, and never produced hypercatharsis or any distressing symptom; on the contrary, it seemed to have rather a soothing and sedative effect. In no case did the dysenteric symptoms continue after salivation was fully established; and rapid convalescence, a voracious appetite, and renewed vigour quickly succeeded the mercurial influence. It is but justice to remark, that the above plan was pursued from the perusal of Mr. Johnson's invaluable work on "the Influence of Tropical Climates on European Constitutions," where the fidelity of description is only equalled by the energy and efficacy of practice recommended.

The causes of these dysenteric attacks were clearly traced to exposure to cold and the damp night air, after working hard in the heat of the day. The febrile symptoms were not, except in one case, where venæsection was employed, in proportion to the local affection of the bowels; but there was more or less of pyrexia in every case. Nothing like contagion was observable.

---

### Mr. Mortimer's *Report of the Yellow or Bulam Fever.*

